# Assessment of proteolytic degradation of the basement membrane: a fragment of type IV collagen as a biochemical marker for liver fibrosis

**DOI:** 10.1186/1755-1536-4-22

**Published:** 2011-10-05

**Authors:** Sanne S Veidal, Morten A Karsdal, Arkadiusz Nawrocki, Martin R Larsen, Yueqin Dai, Qinlong Zheng, Per Hägglund, Ben Vainer, Helene Skjøt-Arkil, Diana J Leeming

**Affiliations:** 1 Nordic Bioscience A/S, Herlev Hovedgade 207, DK-2730 Herlev, Denmark; 2 The Faculty of Health Science, University of Southern Denmark, Campusvej 55, DK-5230 Odense, Denmark; 3 Nordic Bioscience Beijing, No.29 Life Park Road, Zhongguancun Life Science Park, Changping District, Beijing 102206, China; 4 Department of Systems Biology, Technical University of Denmark, Anker Engelunds Vej 1, 2800 Kgs. Lyngby, Denmark; 5 Department of Pathology, Rigshospitalet, University of Copenhagen, Blegdamsvej 9, 2100 Copenhagen, Denmark

**Keywords:** biochemical marker, type IV collagen, neoepitope, basement membrane, extracellular matrix, liver fibrosis, protease-cleaved fragment, matrix metalloproteinase 9

## Abstract

**Background:**

Collagen deposition and an altered matrix metalloproteinase (MMP) expression profile are hallmarks of fibrosis. Type IV collagen is the most abundant structural basement membrane component of tissue, which increases 14-fold during fibrogenesis in the liver. Proteolytic degradation of collagens by proteases produces small fragments, so-called neoepitopes, which are released systemically. Technologies investigating MMP-generated fragments of collagens may provide more useful information than traditional serological assays that crudely measure total protein. In the present study, we developed an ELISA for the quantification of a neoepitope generated by MMP degradation of type IV collagen and evaluated the association of this neoepitope with liver fibrosis in two animal models.

**Methods:**

Type IV collagen was degraded *in vitro *by a variety of proteases. Mass spectrometric analysis revealed more than 200 different degradation fragments. A specific peptide sequence, 1438'GTPSVDHGFL'1447 (CO4-MMP), in the α1 chain of type IV collagen generated by MMP-9 was selected for ELISA development. ELISA was used to determine serum levels of the CO4-MMP neoepitope in two rat models of liver fibrosis: inhalation of carbon tetrachloride (CCl_4_) and bile duct ligation (BDL). The levels were correlated to histological findings using Sirius red staining.

**Results:**

A technically robust assay was produced that is specific to the type IV degradation fragment, GTPSVDHGFL. CO4-MMP serum levels increased significantly in all BDL groups compared to baseline, with a maximum increase of 248% seen two weeks after BDL. There were no changes in CO4-MMP levels in sham-operated rats. In the CCl_4 _model, levels of CO4-MMP were significantly elevated at weeks 12, 16 and 20 compared to baseline levels, with a maximum increase of 88% after 20 weeks. CO4-MMP levels correlated to Sirius red staining results.

**Conclusion:**

This ELISA is the first assay developed for assessment of proteolytic degraded type IV collagen, which, by enabling quantification of basement membrane degradation, could be relevant in investigating various fibrogenic pathologies. The CO4-MMP degradation fragment was highly associated with liver fibrosis in the two animal models studied.

## Background

Liver fibrosis due to viral or alcohol-induced injury is one of the leading causes of death worldwide [1]. To date, no curative treatment for liver fibrosis is available, and patients are dependent on the success of inactivation or removal of the injurious agent or, in the case of end-stage cirrhosis, on liver transplantation. Assessment of liver fibrosis is important to estimate the prognosis for patients with liver cirrhosis and to determine surveillance strategies. At present, liver biopsy is the most common method used to assess fibrosis, but it is invasive and associated with patient discomfort and, in rare cases, serious complications [2]. In addition, the accuracy of liver biopsy is limited because of sampling error and significant intra- and interobserver variability in histological staging [3,4]. Therefore, research has focused on the evaluation of noninvasive methods for the assessment of liver fibrosis [5].

The process leading to liver fibrosis resembles the process of wound healing, including the three phases following tissue injury: inflammation, synthesis of collagenous and noncollagenous extracellular matrix (ECM) components, and tissue remodeling.

Basement membranes in tissues are thin, sheetlike extracellular structures that provide tissue structure and influence cell behavior. The basement membrane is composed of several proteins, such as type IV collagen, laminin, proteoglycans and entactin/nidogen [6]. The presence and distribution of these proteins vary between different tissues. During the pathogenesis of liver fibrosis, the basement membrane components collagen type IV, entactin and laminin increase and form a basement membrane-like structure within the space of Disse [6].

Type IV collagen is the most abundant structural basement membrane component. It consists of a major triple-helix flanked by an N-terminal 7S domain and a C-terminal noncollagenous domain, NC1. There are six distinct type IV collagen chains, α1-α6(IV), that display tissue-specific distribution patterns. Three α chains fold to form a heterotrimeric molecule called a protomer [7]. Remarkably, the six genetically distinct α chains assemble to form only three protomers: α1α1α2(IV), α3α4α5(IV) and α5α5α6(IV). The major ubiquitous form of type IV collagen, α1α1α2(IV), is present in the basement membranes of all tissues, whereas the other two protomers display a more restricted pattern of distribution. Once the protomer is secreted, a complex set of interactions takes place between protomers, forming a collagen type IV network in the shape of a lattice. The important structural role of collagen type IV is illustrated by the clinical consequences of collagen IV mutations, such as Alport syndrome [8,9].

Endopeptidases such as matrix metalloproteinases (MMPs) play a major part in the degradation of extracellular macromolecules such as collagens, and during fibrogenesis, the levels of MMPs increase [10,11]. With respect to excessive proteolytic activity in the fibrous tissue, the gelatinase MMP-9 has been investigated and documented to be highly regulated [10,11].

As a consequence of tissue turnover caused by fibrosis, there is a systemic release of several protein degradation fragments specific for the combination of the involved proteases (such as MMP, the affected organ and the disease). The fragmentation results in the exposure of new peptide ends (so-called neoepitopes), to which specific antibodies can be developed. These neoepitopes may be useful molecular biochemical markers [12].

We hypothesized that it is possible during liver fibrogenesis to measure serum type IV collagen that has been degraded by a specific MMP (MMP-9). The aim of this work was to develop a novel, competitive ELISA for measuring MMP-9-mediated turnover of type IV collagen and to measure the neoepitope 1438'GTPSVDHGFL'1447 (CO4-MMP) in two complementary experimental models of liver fibrosis induced by bile duct ligation (BDL) or carbon tetrachloride (CCl_4_).

## Materials and methods

### Reagents

All reagents used for the experiments were standard high-quality chemicals obtained from companies such as Merck (Whitehouse Station, NJ, USA) and Sigma-Aldrich (St Louis, MO, USA). The synthetic peptides used for monoclonal antibody production were purchased from the Chinese Peptide Company, Beijing, China.

### *In vitro *cleavage

Purified type IV collagen from human placenta (cat. no. 11-511-248457; GenWay Biotech, Inc., San Diego, CA, USA) was cleaved with pro-MMP-2 (cat. no. 444213; Calbiochem, Gibbstown, NJ, USA) or pro-MMP-9 (cat. no. 444231; Merck, Whitehouse Station, NJ, USA). Fifty micrograms of MMP-2 or MMP-9 were activated with 20 μL of 1 mM 4-aminophenylmercuric acetate in dimethyl sulfoxide and incubated at 37°C for three hours. Type IV collagen was delivered dissolved in 0.5 M acetic acid. To facilitate MMP cleavage, the protein was dialyzed for two days to remove the acetic acid. The liquid was filtered to remove proteins below 10 kDa (cat. no. 42407, Microcon Ultracel YM-10; Millipore, Billerica, MA, USA). Each MMP cleavage was performed separately by mixing 100 μg of type IV collagen and 10 μg of either MMP-2 or MMP-9 in MMP buffer (100 mM Tris·HCl, 100 mM NaCl, 10 mM CaCl_2_, 2 mM Zn acetate, pH 8.0). As a control, 100 μg of collagen was mixed with MMP buffer alone. The solutions were incubated for two hours at 37°C. The cleavage reaction was stopped using 50 μM ethylenediaminetetraacetic acid (EDTA) to a final concentration of 1 μM. Cleavage was verified by visualization using the SilverXpress Silver Staining Kit (cat. no. LC6100; Invitrogen, Carlsbad, CA, USA) according to the manufacturer's instructions.

### Peptide identification

Peptide fragments in the *in vitro *cleaved samples were identified using liquid chromatography (LC) coupled to electrospray ionization (ESI) tandem mass spectrometry (LC-MS/MS). LC-MS samples were ultrafiltrated to remove proteins above 10 kDa, the pH was adjusted to 2.0 using formic acid and a 4 μL sample was analyzed by LC-MS/MS. LC was performed on a nanoACQUITY UPLC BEH C_18 _Column (Waters, Milford, MA, USA) using a formic acid/acetonitrile gradient. MS and MS/MS were performed on a Synapt G1 High Definition Mass Spectrometer quadrupole time-of-flight MS (QUAD-TOF; Waters) with an acquisition range of 350 to 1,600 *m/z *in MS and 50 to 2000 *m/z *in MS/MS. ProteinLynx Global SERVER software (Waters) was used to analyze spectra and generate peak lists. To identify peptides, MS and MS/MS data were searched against a type IV collagen protein database (FASTA) using Mascot 2.2 software (Matrix Science, Boston, MA, USA) with the ESI-QUAD-TOF settings and carbamidomethyl (C), oxidation of methionine (M), oxidation of lysine (K) and oxidation of proline (P) as variable modifications.

The six amino acids in the N- or C-terminal of the peptides identified by MS were regarded as a neoepitope generated by the protease in question. All protease-generated sequences were analyzed for homology and distance to other cleavage sites and tested for homology using NPS@:network protein sequence analysis [13].

### Peptide conjugation

The peptide conjugation was performed using the Maleimide Activated BSA, KLH Conjugation Kit (Sigma-Aldrich). Briefly, the cysteine-containing immunogenic neoepitope (CGG-GTPSVDHGFL, 400 μL of peptide at 5 mg/mL) with one free sulfhydryl group (-SH) was mixed in conjugation buffer containing the maleimide-activated ovalbumin (OVA) (180 μL of OVA at 10 mg/mL) as a carrier protein with an available maleimide group that could react with -SH-containing peptides and incubated for two hours at room temperature. Conjugated products were cleared of EDTA and sodium azide by desalting or dialysis for two days. The biotin-conjugated lysine was added to the biotin-conjugated peptides in the solid-phase peptide synthesis procedure.

### Monoclonal antibody development

Four- to six-week-old Balb/c mice were immunized subcutaneously with about 200 μL of emulsified antigen and 50 μg of the neoepitope CO4-MMP (OVA-CGG-GTPSVDHGFL). Consecutive immunizations were performed at two-week intervals until stable sera titer levels were reached in Freund's incomplete adjuvant. Blood samples were collected from the second immunization. For each blood sampling, the serum titer was determined and the mouse with the highest antiserum titer was selected for fusion. After the fourth immunization, this mouse was rested for one month and then boosted intravenously with 50 μg of CO4-MMP in 100 μL of 0.9% sodium chloride solution three days before isolation of the spleen for cell fusion.

### Fusion and antibody screening

The fusion procedure was performed as described by Gefter *et al*. [14]. Briefly, mouse spleen cells were fused with SP2/0 myeloma fusion partner cells. The hybridoma cells were cloned using a semisolid medium method, transferred into 96-well microtiter plates for further growth and incubated in a CO_2 _incubator. Standard limited dilution was used to promote monoclonal growth. Supernatants were screened using an indirect ELISA with streptavidin-coated microtiter plates and biotin-CGG-GTPSVDHGFL as a capture peptide.

### Characterization of clones

Native reactivity and peptide binding of the monoclonal antibodies were evaluated by displacement of native samples (human/rat/mouse serum, plasma and urine) in a preliminary ELISA using 10 ng/mL biotinylated peptide coater on a streptavidin-coated microtiter plate and the supernatant from the growing monoclonal hybridoma. Specificities of the clones to a free peptide (GTPSVDHGFL), a non-sense peptide and an elongated peptide (TPSVDHGFLV) were tested. Isotyping of the monoclonal antibodies was performed using the Clonotyping System-HRP kit (cat. no. 5300-05; Southern Biotech, Birmingham, AL, USA). The selected clones were purified using protein G columns according to the manufacturer's instructions (GE Healthcare Life Sciences, Little Chalfont, UK). Selected monoclonal antibodies were labeled with horseradish peroxidase (HRP) using the Lightning-Link Horseradish Peroxidase labeling kit (Innova Biosciences, Cambridge, UK) according to the instructions of the manufacturer.

### CO4-matrix metalloproteinase enzyme-linked immunosorbent assay methodology

In preliminary experiments, we optimized the reagents, their concentrations and the incubation periods by performing several checkerboard analyses. The CO4-MMP ELISA was developed as follows: A 96-well streptavidin plate was coated with biotinylated synthetic peptide biotin-CGG-GTPSVDHGFL, dissolved in PBS buffer (2 mM KH_2_PO_4_, 9 mM Na_2_HPO_4_, 2H_2_O, 3 mM KCl, 137 mM NaCl, pH 7.4) and incubated for 30 minutes at 20°C by constant shaking at 200 rpm. Twenty microliters of peptide calibrator or sample dissolved in assay buffer (25 mM Tris, 1% BSA, 0.1% Tween 20, pH 7.4) were added to appropriate wells, followed by 100 μL of conjugated monoclonal antibody, and incubated for one hour at 20°C by constant shaking at 300 rpm. Finally, 100 μL of tetramethylbenzidine (TMB) (cat. no. 438OH; Kem-En-Tec, Copenhagen, Denmark) were added, and the plate was incubated for 15 minutes at 20°C in the dark and shaken at 300 rpm. After each incubation step, the plate was washed five times in washing buffer (20 mM Tris, 50 mM NaCl, pH 7.2). The TMB reaction was stopped by adding 100 μL of stopping solution (1% HCl) and measured spectrophotometrically at 450 nm with 650 nm as the reference. A standard curve was performed by serial dilution of the CO4-MMP peptide and plotted using a four-parametric mathematical fit model. Standard concentrations were 0, 1.5625, 3.125, 6.25, 12.5, 25, 50 and 100 ng/mL.

### Technical evaluation

From twofold dilutions of pooled serum and plasma samples, linearity was calculated as a percentage of recovery of the 100% sample. The lower detection limit (LDL) was calculated from 21 determinations of the lowest standard (the zero standard) and calculated as the mean 3 × standard deviation. The inter- and intraassay variations were determined by ten independent runs of five QC samples, with each run consisting of two replicates of double-determinations of the samples. Spiking recovery was determined by comparing different concentrations of a peptide sample in buffer and in human serum. The peptide sample was the type IV collagen cleaved with MMP-9 and pepsin. Finally, for each assay, a master calibrator prepared from synthetic peptides accurately quantified by amino acid analysis was used for calibration purposes. The analyte stability was determined for six serum samples (three rat and three human) for ten freeze-thaw cycles.

### Enzyme-linked immunosorbent assay characterization

The developed CO4-MMP ELISA was evaluated using 20 μL of the samples: intact type IV collagen, type IV collagen cleaved with pepsin, type IV collagen cleaved with MMP-2, type IV collagen cleaved with MMP-9, type IV collagen cleaved with MMP-9 and pepsin, and an elongated CO4-MMP amino acid sequence (TPSVDGHFLV). Cross-reactivity was tested using *in vitro *cleaved collagen type I or type VI.

### Bile duct ligation

A total of 81 female Sprague-Dawley rats, age six months, were housed at the animal research facilities at Nordic Bioscience A/S, Herlev, Denmark. The experiments were approved by the Experimental Animal Committee of the Danish Ministry of Justice and were performed according to the European Standard for Good Clinical Practice (2008/561-1450). The rats were housed in standard cages at 18°C to 22°C with bedding and nest material (1324 TPF; Altromin Spezialfutter GmbH & Co. KG, Lage, Germany) and tap water *ad libitum*. The rats were kept under 12-hour light-dark cycle conditions. Experiments began after one week of acclimatization. Liver fibrosis was induced in anesthetized rats by standard BDL in which the bile duct was ligated in two places and dissected between the ligations prior to closing the abdomen. In sham-operated rats, the abdomen was closed without BDL.

The rats were divided into four groups: rats in group 1 (ten BDL and eight sham) were killed after one week, rats in group 2 (12 BDL and 8 sham) were killed after two weeks, rats in group 3 (13 BDL and 8 sham) were killed after three weeks and rats in group 4 (14 BDL and 8 sham) were killed after four weeks.

### Carbon tetrachloride inhalation

The study included 52 three-month-old male Wistar rats treated with CCl_4 _and 28 Wistar control rats (Charles River Laboratories, Saint-Aubin-lès-Elbeuf, France). Complete details of the study are described elsewhere [15]. Liver damage was induced as previously described [16] and, in short, included inhalation of CCl_4 _twice weekly. Phenobarbital (0.3 g/L) was added to the drinking water. Animals were stratified into groups receiving 8, 12, 16 or 20 weeks of CCl_4 _(for each group, n = 13 CCl_4 _and n = 7 control). Control rats received phenobarbital only. After the stated weeks of CCl_4 _administration, the rats were weighed, anesthetized with pentobarbital (50 mg/kg) and killed by decapitation. The study was performed according to the criteria of the Investigation and Ethics Committee of the Hospital Clinic Universitari (Barcelona, Spain).

### Blood and tissue sampling

Blood samples were taken under light CO_2_/O_2 _anesthesia at baseline and at death from the retroorbital sinuses of rats that had been fasted for at least 14 hours. The collected blood was left for 30 minutes at room temperature to clot, followed by centrifugation at 3,000 *g *for 10 minutes. All clot-free liquid was transferred to new tubes and centrifuged again at 3,000 *g *for 10 minutes. The serum was then transferred to clean tubes and stored at -80°C.

The livers were carefully dissected, weighed, fixed in 4% formaldehyde for a minimum of 24 hours, cut into appropriate slices and embedded in paraffin. Liver sections (4 to 5 μm thick) were stained with 0.1% Sirius red F3B (Sigma-Aldrich) in saturated picric acid (Sigma-Aldrich).

### Histological image analysis

Relative fibrosis area (expressed as a percentage of total liver area) was assessed by analyzing 36 fields of Sirius red-stained liver sections per animal. Each field was acquired at ×10 magnification using an E600 microscope (Nikon Instruments Inc., Melville, NY, USA) equipped with a SPOT RT Slider CCD digital camera (Diagnostic Instruments, Inc., Sterling Heights, MI, USA). The results were analyzed using a computerized Bioquant Life Science morphometric software system (Bioquant Image Analysis Corp., Nashville, TN, USA. To evaluate the relative fibrosis area, the measured collagen area was divided by the net field area and then multiplied by 100. Subtraction of vascular luminal area from the total field area yielded the final calculation of the net fibrosis area. For each animal analyzed, the amount of fibrosis was measured as a percentage and the average value is presented [17].

### Immunohistochemistry

Liver sections (4 to 5 μm) were deparaffinized and hydrated, and further peroxidase activity was blocked with the addition of 0.4% hydrogen peroxide. Sections were then incubated with a polyclonal antibody against type IV collagen (1:100; Abcam, Cambridge, UK). Sections were then rinsed, and the antibody binding was depicted using the Super Sensitive Polymer-HRP IHC Detection System combined with AEC substrate according to the supplier's instructions (Biogenex, Taby, Sweden). Sections were counterstained with Mayer's hematoxylin. Digital photographs were taken using an Olympus B 60 × microscope at ×40 magnification equipped with an Olympus C-5050 Zoom digital camera (Olympus, Tokyo, Japan).

### Statistical analysis

Mean values and standard errors of the mean (SEM) were calculated using GraphPad Prism software (GraphPad Software, San Diego, CA, USA), and statistical significance was assessed using the Student's two-tailed paired *t*-test (α = 0.05) assuming normal distribution or the Mann-Whitney two-tailed nonparametric *U *test (α = 0.05). The coefficient of correlation (*R*^2^) and the corresponding *P *values were determined by linear regression.

## Results

### *In vitro *cleavage and selection of peptides

Fragments of type IV collagen cleaved by MMP-2 or MMP-9 with a statistically significant Mascot score (*P *< 0.05) were identified. All protease-generated neoepitopes were tested for homology. Among more than 200 MMP-generated neoepitopes, five sequences were selected for immunization, as these sequences are unique to type IV collagen. In addition, the sequences 3 to 5 were found in various animal species. The five sequences selected were (1) 328'PGPPGIVIGT.'337, CO4a1 generated by MMP-9; (2) 333'.IVIGTGPLGE'342, CO4a1 generated by MMP-9; (3) 777'.LQGIRGEPGP'786, CO4a1 generated by MMP-9; (4) 1330'DQGDQGVPGA'1339, CO4a1 generated by MMP-2 or MMP-9; and 1438'GTPSVDHGFL'1447, CO4a1 generated by MMP-2 or MMP-9.

The sequence 1438'GTPSVDHGFL'1447 (CO4-MMP) in the α1 chain of type IV collagen generated by MMP-2 or MMP-9 was selected as having the best technical performance because the antibodies were able to distinguish between cleaved and uncleaved type IV collagen. This sequence was 100% homologous in human, rat and mouse.

### Clone characterization

**Figure 1:**
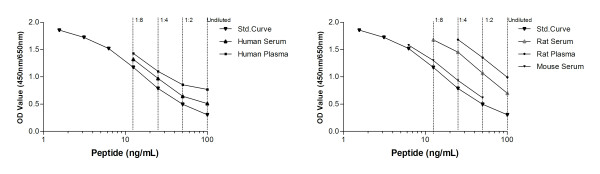
**Standard curves**. ELISA run showing typical standard curves and native reactivity against (A) Human serum, plasma, and (B) Rodents: rat serum and plasma and mouse serum. Native material was run undiluted and at 1:2, 1:4 and 1:8 dilutions as indicated by dashed lines. The signal is the optical density at 450 nm, subtracting the background at 650 nm, as a function of peptide concentration.

After the fusion between spleen cells and myeloma cells, seven hybridoma cell lines remained. After comparing native reactivity, antibody affinity and stability, the best antibody-producing cell line was chosen. The clone selected was the immunoglobulin G2a subtype. The antibody was reactive to human, rat and mouse plasma and serum (Figure 1).

### Technical evaluation

**Figure 2:**
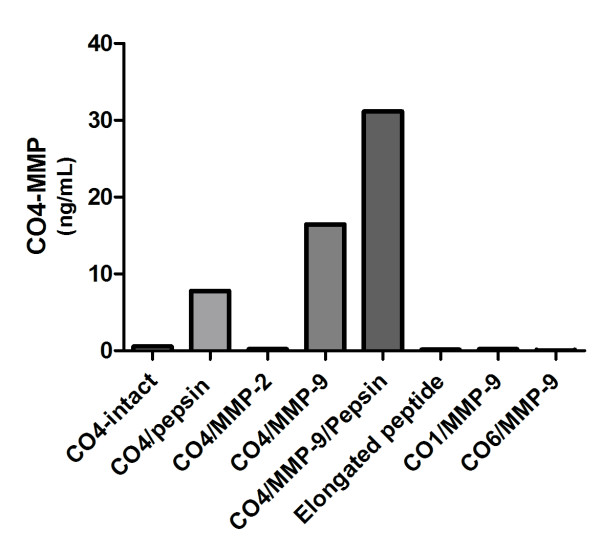
**Assay characterization**. Characterization of the 1438'GTPSVDHGFL'1447 (CO4-MMP) assay with regard to reactivity against intact type IV collagen (CO4 intact), type IV collagen cleaved by pepsin (CO4/pepsin), type IV collagen cleaved by matrix metalloproteinase 2 (CO4/MMP-2), type IV collagen cleaved by MMP-9 (CO4/MMP-9), type IV collagen cleaved by MMP-9 and pepsin, elongated peptide with extension of one amino acid at the neoepitope site, type I collagen cleaved by MMP-9 (CO1/MMP-9) and type VI collagen cleaved by MMP-9 (CO6/MMP-9).

The typical standard curve is presented in Figure 2, which shows a four-parametric fit for the assay. The LDL for the CO4-MMP assay was 0.61 ng/ml. Dilution recovery was within 100% ± 20% (Table 1) for human and rat serum. The mean intra- and interassay variations were 4.8% and 12.1%, respectively (Table 2). Analyte stability was acceptable for 2 × to 10 × freeze-thaw cycles for both rat and human serum (Table 3). The percentages are within 100 ± 20%, except for one determination at 8 × for human serum and one determination at 6 × for rat serum. Finally, spiking recovery of peptide in human serum was acceptable, with recoveries ranging from 103% to 110% (Table 4).

**Table 1:** Percentage dilution recovery for the CO4-MMP assay^a^

CO4-MMP, ng/mL	HS1 63.0	HS2 73.7	RS1 42.2	RS2 38.4
Undiluted	100%	100%	100%	100%
Dilution 1:2	92.4%	99.6%	116.1%	108.7%
Dilution 1:4	101.5%	90.4%	123.7%	120.3%
Dilution 1:8	95.8%	91.5%	120.7%	126.9%
Dilution 1:16	80.4%	81.9%	117.5%	122.2%
Mean	92.5%	90.9%	119.5%	119.5%

^a^CO4-MMP = 1438'GTPSVDHGFL'1447; HS(1 or 2) = human serum samples; RS(1 or 2) = rat serum samples. The percentages represents difference from a undiluted sample.

**Table 2:** Inter- and intraassay variation for the CO4-MMP assays using human serum quality control samples^a^

CO4-MMP sample	Amount, ng/mL	Intraassay variability, %	Interassay variability, %
HS1	28.64	5.2	16.3
HS2	35.24	6.7	13.5
HS3	39.29	5.9	11.1
HS4	42.54	4.6	9.6
HS5	47.12	4.7	15.0
HS6	51.94	2.9	12.6
HS7	51.77	2.9	14.1
HS8	48.93	5.1	11.2
Mean		4.8	12.1

^a^CO4-MMP = 1438'GTPSVDHGFL'1447; The variation was calculated as the mean variation of ten individual determinations for each sample.

**Table 3:** Analyte stability in three rat and three human serum samples in ten freeze-thaw cycles^a^

Analyte stability	Percentage recovery compared to 1 × freeze-thaw cycle	
Freeze-thaw cycles	Human CO4-MMP	Rat CO4-MMP
2 ×	109.9	81.6
3 ×	75.4	88.9
4 ×	95.6	101.8
5 ×	113.1	85.3
6 ×	92.2	71.1
7 ×	122.6	92.1
8 ×	131.0	83.6
9 ×	104.3	91.0
10 ×	90.9	96.6

^a^All data shown are percentage recovery compared to day 0 or day 1 freeze-thaw cycle.

**Table 4:** Spiking recovery in human serum^a^

Peptide concentration, ng/mL	Peptide serum + peptide concentration, ng/mL	Peptide recovered, ng/mL	Recovery, %
31.1	145.3	31.1	103.5
13.4	135.3	13.3	110.4
7.6	125.7	7.6	107.6
4.7	121.6	4.7	106.7
0	109.2	0	100

^a^Peptide sample = native type IV collagen cleaved by matrix metalloproteinase 9 and pepsin. Recovery percentages are peptide concentrations compared to peptide measured in serum after subtracting the serum background.

### Enzyme-linked immunosorbent assay characterization

To characterize the analytes detected in the assay, different collagens were cleaved with different proteases. Pepsin or MMP-9 alone or together was able to generate the CO4-MMP fragment from collagen type IV alone (Figure 2). In contrast, the fragment was not found in intact type IV collagen or MMP-2 cleaved type IV collagen. Finally, no cross-reactivity was seen between CO4-MMP and MMP-9 cleaved type I or VI collagens, which have high homology with the immunization sequence of type IV collagen. No reactivity was seen against the elongated synthetic peptide, proving neoepitope reactivity (Figure 2).

### Evaluations performed in the bile duct ligation study

**Figure 3:**
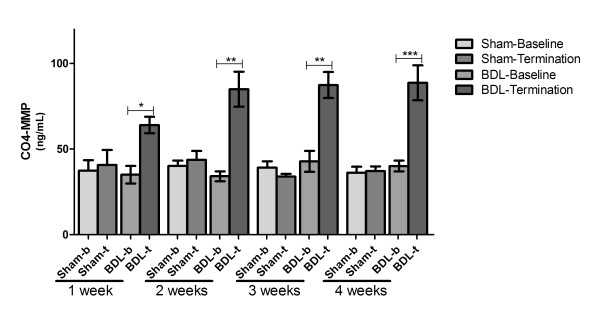
**CO4-MMP serum levels in bile duct-ligated rats**. Serum 1438'GTPSVDHGFL'1447 (CO4-MMP) was assessed in bile duct-ligated and sham-operated rats at baseline and at death at weeks 1, 2, 3 and 4. The data presented are means ± standard errors of the mean (SEM). Asterisks indicate statistical significance as indicated by bars (**P < 0.05, ***P < 0.001, ns = nonsignificant difference).

During the four-week experiment, 15 of 81 rats, 14 of them BDL-operated, were killed because of excessive weight loss. CO4-MMP serum levels increased significantly in all BDL groups compared with baseline (group 1: baseline 35.1 ng/mL, death 64.1 ng/mL, *P *< 0.05; group 2: baseline 34.2 ng/mL, death 84.9 ng/mL, *P *< 0.01; group 3: baseline 42.9 ng/mL, death 87.4 ng/mL, *P *< 0.01; group 4: baseline 40.1 ng/mL, death 88.7 ng/mL, *P *< 0.001), with the maximum increase from baseline of 248% seen in group 2. CO4-MMP levels did not change significantly in the sham-operated rats (Figure 3).

### Evaluations performed in the carbon tetrachloride study

**Figure 4:**
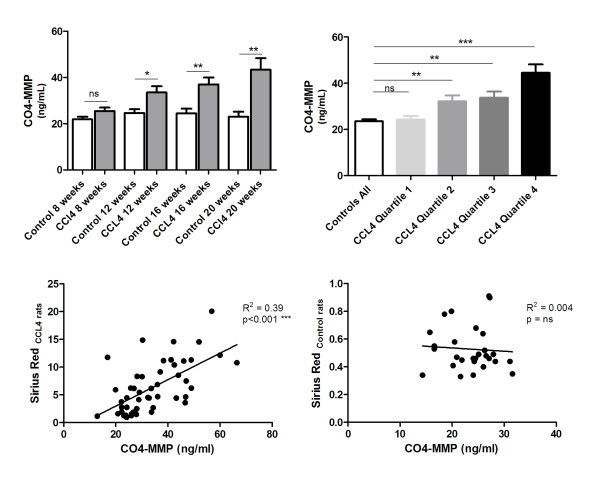
**CO4-MMP serum levels in carbon tetrachloride-treated rats**. (A) Serum 1438'GTPSVDHGFL'1447 (CO4-MMP) was assessed in control rats at death (controls) as well as in carbon tetrachloride (CCl_4_)-treated rats at death at weeks 8, 12, 16 and 20. The results shown are means ± SEM. (B) Serum CO4-MMP in all controls pooled and CCl_4 _rats stratified into quartiles according to total collagen in the liver. (C) Correlations between CO4-MMP and Sirius red in (C) CCl_4 _rats and (D) control rats. Asterisks indicate statistical significance as indicated by bars (**P < 0.05, ***P < 0.001, ns = nonsignificant difference).

**Figure 5:**
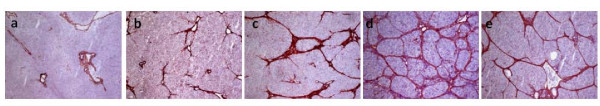
**Type IV collagen in the liver**. Immunohistochemical analysis of type IV collagen. Photomicrographs show hepatic structure in rats 20 weeks after vehicle treatment (a), after 8 weeks of carbon tetrachloride (CCl_4_) treatment (b), after 12 weeks of CCl_4 _treatment (c), after 16 weeks of CCl_4 _treatment and (d) and after 20 weeks of CCl_4 _treatment. Original magnification, ×40.

Four rats in the CCl_4 _groups died during the study. Among the surviving animals, levels of CO4-MMP were significantly elevated at all time points compared to baseline levels, except at week 8 (week 12: control 24.6 ng/mL, CCl_4 _33.5 ng/mL, *P *< 0.05; week 16: control 24.5 ng/mL, CCl_4 _36.9 ng/mL, *P *< 0.01; week 20: control 23.0 ng/mL, CCl_4 _43.3 ng/mL, *P *< 0.01) (Figure 4). The immunohistochemistry showed that collagen type IV deposition was exclusively in the venous wall and around the space of Disse in control rats (Figure 5a). In contrast, in CCl_4_-treated rats, type IV collagen was located along the fibrotic bands (Figures 5b through 5e).

## Discussion

To the best of our knowledge, this study is the first to present the development of an assay specific for a fragment generated by the degradation of type IV collagen by MMPs. This fragment may be a useful marker for degradation of the basement membrane in tissue.

Type IV collagen is the most abundant structural basement membrane component of tissue, with an increase of 14-fold during fibrogenesis in the liver, which is the highest relative increase among collagens [18]. Several assays already exist for measuring total type IV collagen in different pathologies by using polyclonal or monoclonal antibodies. Type IV collagen has been studied in hepatitis C and nonalcoholic fatty liver disease, and adequate diagnostic performance for significant fibrosis has been reported, particularly in hepatitis C [19,20].

In our study, protease cleavage was performed by incubating type IV collagen with various MMPs and control proteases, after which fragments were identified by MS analysis. Type IV collagen degradation resulted in many unique fragments, among which was the CO4-MMP fragment (GTPSVDHGFL). We consequently developed a novel, technically robust ELISA with monoclonal antibodies to measure CO4-MMP (GTPSVDHGFL) in serum. The assay had acceptable inter- and intraassay variation, dilution recovery, spiking recovery and LDL.

CO4-MMP levels were assessed in two different animal models of liver fibrosis: CCl_4 _and BDL. In both models, we found significantly increased CO4-MMP levels in rats with fibrotic livers compared with controls. In addition, CO4-MMP levels were significantly correlated with collagen deposition in the CCl_4 _model of liver fibrosis. The data are in alignment data reported in other studies in these animal models, demonstrating that the presence of neoepitopes is a good biochemical marker of pathologies involving excessive ECM remodeling [21-27]. A difference in the levels of CO4-MMP in the BDL and CCl_4 _control/sham groups was observed, which we attribute to the differences in age, sex and strain of rats used. The animals in the CCl_4 _study were three-month-old male Wistar rats, whereas BDL animals were six-month-old female Sprague-Dawley rats.

It is well appreciated that the BDL and the CCl_4 _models describe two different fibrotic processes, although increased ECM remodeling and excessive collagen deposition are key characteristics of both. CO4-MMP levels were significantly related to liver fibrosis in rats treated with CCl_4 _for 12 to 20 weeks. The marker also correlated highly significantly to total collagen in the livers of CCl_4_-treated rats, albeit not in the control rats, strongly indicating the fibrosis-specific pathological relevance of the neoepitope. In the BDL model of liver fibrosis, serum CO4-MMP was elevated one week after BDL surgery compared to baseline and sham levels. The serum CO4-MMP levels remained significantly increased in rats studied for up to four weeks. These data are in agreement with data reported in previous studies, highlighting that type IV collagen is generated during liver fibrogenesis by the activated hepatic stellate cells in the liver [28] and that MMP levels become elevated and imbalanced during fibrosis [1,29]. These data support the hypothesis that liver fibrosis results not simply from an increase in collagen formation, but rather from an increase in both collagen degradation and formation, with formation outpacing degradation and leading to a net increase.

The presently reported data clearly demonstrate that the ELISA measured exclusively degraded type IV collagen, by MMP-9 or pepsin alone or together, and not fibrillar collagens types I, II and III. Future detailed examination of the precise analyte we identified for our CO4-MMP assay may shed more light on the processes leading not only to tissue degradation but also to tissue formation. The current assay provides additional information compared to that of total collagen assessment, as this assessment exclusively detects type IV collagen degradation in the face of a pool of type IV collagen fragments of both formation and degradation, which may better be considered turnovers markers

This study carries some limitations. One major limitation of this study is that it was carried out in homogeneous, inbred laboratory rats with synchronous induction of liver disease, which bears little resemblance to the highly complicated clinical presentation of liver fibrosis. Further investigations in clinical settings are needed to provide more information on the usefulness of the CO4-MMP biomarker.

The recent cloning and characterization of genes for collagen IV have provided information on the structure, expression and function of basement membranes during development and diseases states. The important role of collagen type IV is illustrated by the clinical consequences of collagen IV mutations leading to, for example, Alport syndrome caused by mutations in the α3 chain, HANAC syndrome (hereditary angiopathy with nephropathy, aneurysm and cramps) caused by mutations in the α1 chain, defects in Bowman's capsule consisting of hypertrophy of parietal epithelium caused by α1 mutations, anterior segment dysgenesis caused by α1 mutations and Axenfeld syndrome caused by α5 mutations [8,9].

Type IV collagen forms a complex with many interactions, including intrachain covalent bonds, as well as interactions with most other constituents of the basement membrane [6]. Thus, measurement of type IV collagen may be different compared to the assays developed for measurement of degradation of the fibrillar collagens types I, II and III [21,30-33]. The present data clearly demonstrate that the ELISA measured exclusively degraded type IV collagen. Others have used pepsin-solubilized type IV collagen to assess serum levels [34], as the protein is caught in a complex. The CO4-MMP fragment was identified by MS data analysis as being generated by MMP-2 and MMP-9, but only the MMP-9-generated fragment was detected by the CO4-MMP assay. Type IV collagen may be cleaved without release of fragments that are bound to a multivalent complex. This explains why we observed a stronger reaction when we cleaved first with MMP-9 and then with pepsin. Further research is needed to understand which enzymes and their sequential activities are involved in the degradation process.

## Conclusions

In summary, we have developed an assay using a specific monoclonal antibody for the detection of CO4-MMP, a basement membrane degradation marker. We have demonstrated that this marker was elevated in two preclinical models of liver fibrosis, the BDL and the CCl_4 _rat models, indicating that this neoepitope may have potential use in assessing diseases with high turnover of the ECM.

## Abbreviations

BDL: bile duct ligation; BSA: bovine serum albumin; CCl_4_: carbon tetrachloride; ELISA: enzyme-linked immunosorbent assay; ECM: extracellular matrix; HS: human serum; LC-MS: liquid chromatography-mass spectrometry; LDL: lower detection limit; MMP: matrix metalloproteinase; ns: nonsignificant; PBS: phosphate-buffered saline; RS: rat serum; SEM: standard error of the mean.

## Competing interests

SSV, MAK, YD, QZ, HBE, and DJL are employees of Nordic Bioscience A/S. MAK owns stocks and shares in Nordic Bioscience A/S.

## Authors' contributions

SSV designed the study, developed the CO4-MMP assay, performed histology and participated in manuscript drafting. AN, MRL and PH helped with the MS analysis and identification of type IV collagen fragments. QZ, YD and HSA participated in the assay development. BV participated in the study design and discussed data analysis. MAK and DJL participated in the study design, discussed data analysis and participated in manuscript drafting. All authors read and approved the final manuscript.

## References

[B1] Friedman SL Liver fibrosis: from bench to bedside J Hepatol 2003 4 Suppl 1 S38 S53 12591185 10.1016/s0168-8278(02)00429-4

[B2] Gressner OA Weiskirchen R Gressner AM Biomarkers of liver fibrosis: clinical translation of molecular pathogenesis or based on liver-dependent malfunction tests Clin Chim Acta 2007 4 107 113 10.1016/j.cca.2007.02.038 17399697

[B3] Bedossa P Dargère D Paradis V Sampling variability of liver fibrosis in chronic hepatitis C Hepatology 2003 4 1449 1457 14647056 10.1016/j.hep.2003.09.022

[B4] Maharaj B Maharaj RJ Leary WP Cooppan RM Naran AD Pirie D Pudifin DJ Sampling variability and its influence on the diagnostic yield of percutaneous needle biopsy of the liver Lancet 1986 4 523 525 2869260 10.1016/s0140-6736(86)90883-4

[B5] Veidal SS Bay-Jensen AC Tougas G Karsdal MA Vainer B Serum markers of liver fibrosis: combining the BIPED classification and the neo-epitope approach in the development of new biomarkers Dis Markers 2010 4 15 28 20164543 10.3233/DMA-2010-0678 PMC3833336

[B6] Rowe RG Weiss SJ Breaching the basement membrane: who, when and how? Trends Cell Biol 2008 4 560 574 10.1016/j.tcb.2008.08.007 18848450

[B7] Hudson BG Reeders ST Tryggvason K Type IV collagen: structure, gene organization, and role in human diseases: molecular basis of Goodpasture and Alport syndromes and diffuse leiomyomatosis J Biol Chem 1993 4 26033 26036 8253711

[B8] Gubler MC Inherited diseases of the glomerular basement membrane Nat Clin Pract Nephrol 2008 4 24 37 10.1038/ncpneph0671 18094725

[B9] Van Agtmael T Bruckner-Tuderman L Basement membranes and human disease Cell Tissue Res 2010 4 167 188 10.1007/s00441-009-0866-y 19756754

[B10] Hemmann S Graf J Roderfeld M Roeb E Expression of MMPs and TIMPs in liver fibrosis: a systematic review with special emphasis on anti-fibrotic strategies J Hepatol 2007 4 955 975 10.1016/j.jhep.2007.02.003 17383048

[B11] Kirimlioglu H Kirimlioglu V Yilmaz S Expression of matrix metalloproteinases 2 and 9 in donor liver, cirrhotic liver, and acute rejection after human liver transplantation Transplant Proc 2008 4 3574 3577 10.1016/j.transproceed.2008.09.033 19100442

[B12] Karsdal MA Henriksen K Leeming DJ Mitchell P Duffin K Barascuk N Klickstein L Aggarwal P Nemirovskiy O Byrjalsen I Qvist P Bay-Jensen AC Dam EB Madsen SH Christiansen C Biochemical markers and the FDA Critical Path: how biomarkers may contribute to the understanding of pathophysiology and provide unique and necessary tools for drug development Biomarkers 2009 4 181 202 10.1080/13547500902777608 19399662

[B13] Combet C Blanchet C Geourjon C Deléage G NPS@: network protein sequence analysis Trends Biochem Sci 2000 4 147 150 10.1016/S0968-0004(99)01540-6 10694887

[B14] Gefter ML Margulies DH Scharff MD A simple method for polyethylene glycol-promoted hybridization of mouse myeloma cells Somatic Cell Genet 1977 4 231 236 10.1007/BF01551818 605383

[B15] Segovia-Silvestre T Reichenbach V Fernández-Varo G Vassiliadis E Barascuk N Morales-Ruiz M Karsdal MA Jiménez W Circulating CO3-610, a degradation product of collagen III, closely reflects liver collagen and portal pressure in rats with fibrosis Fibrogenesis Tissue Repair 2011 4 19 10.1186/1755-1536-4-19 21813019 PMC3170588

[B16] Clariá J Jiménez W Renal dysfunction and ascites in carbon tetrachloride-induced cirrhosis in rats In The Liver and the Kidney 1999 Boston: Blackwell Science 379 396

[B17] Muñoz-Luque J Ros J Fernández-Varo G Tugues S Morales-Ruiz M Alvarez CE Friedman SL Arroyo V Jiménez W Regression of fibrosis after chronic stimulation of cannabinoid CB2 receptor in cirrhotic rats J Pharmacol Exp Ther 2008 4 475 483 18029545 10.1124/jpet.107.131896 PMC2887659

[B18] Gressner AM Weiskirchen R Modern pathogenetic concepts of liver fibrosis suggest stellate cells and TGF-β as major players and therapeutic targets J Cell Mol Med 2006 4 76 99 10.1111/j.1582-4934.2006.tb00292.x 16563223 PMC3933103

[B19] Murawaki Y Koda M Okamoto K Mimura K Kawasaki H Diagnostic value of serum type IV collagen test in comparison with platelet count for predicting the fibrotic stage in patients with chronic hepatitis C J Gastroenterol Hepatol 2001 4 777 781 10.1046/j.1440-1746.2001.02515.x 11446886

[B20] Walsh KM Fletcher A MacSween RN Morris AJ Basement membrane peptides as markers of liver disease in chronic hepatitis C J Hepatol 2000 4 325 330 10.1016/S0168-8278(00)80079-3 10707874

[B21] Barascuk N Veidal SS Larsen L Larsen DV Larsen MR Wang J Zheng Q Xing R Cao Y Rasmussen LM Karsdal MA A novel assay for extracellular matrix remodeling associated with liver fibrosis: an enzyme-linked immunosorbent assay (ELISA) for a MMP-9 proteolytically revealed neo-epitope of type III collagen Clin Biochem 2010 4 899 904 10.1016/j.clinbiochem.2010.03.012 20380828

[B22] Vassiliadis E Veidal SS Simonsen H Larsen DV Vainer B Chen X Zheng Q Karsdal MA Leeming DJ Immunological detection of the type V collagen propeptide fragment, PVCP-1230, in connective tissue remodeling associated with liver fibrosis Biomarkers 2011 4 426 433 10.3109/1354750X.2011.584131 21612338

[B23] Veidal SS Vassiliadis E Bay-Jensen AC Tougas G Vainer B Karsdal MA Procollagen type I N-terminal propeptide (PINP) is a marker for fibrogenesis in bile duct ligation-induced fibrosis in rats Fibrogenesis Tissue Repair 2010 4 5 10.1186/1755-1536-3-5 20359335 PMC2860343

[B24] Veidal SS Vassiliadis E Barascuk N Zhang C Segovia-Silvestre T Klickstein L Larsen MR Qvist P Christiansen C Vainer B Karsdal MA Matrix metalloproteinase-9-mediated type III collagen degradation as a novel serological biochemical marker for liver fibrogenesis Liver Int 2010 4 1293 1304 10.1111/j.1478-3231.2010.02309.x 20666994

[B25] Leeming DJ Larsen DV Zhang C Hi Y Veidal SS Nielsen RH Henriksen K Zheng Q Barkholt V Riis BJ Byrjalsen I Qvist P Karsdal MA Enzyme-linked immunosorbent serum assays (ELISAs) for rat and human N-terminal pro-peptide of collagen type I (PINP): assessment of corresponding epitopes Clin Biochem 2010 4 1249 1256 10.1016/j.clinbiochem.2010.07.025 20709044

[B26] Vassiliadis E Larsen DV Clausen RE Veidal SS Barascuk N Larsen L Simonsen H Silvestre TS Hansen C Overgaard T Leeming DJ Karsdal MA Measurement of CO3-610, a potential liver biomarker derived from matrix metalloproteinase-9 degradation of collagen type iii, in a rat model of reversible carbon-tetrachloride-induced fibrosis Biomark Insights 2011 4 49 58 21499440 10.4137/BMI.S6347 PMC3076019

[B27] Vassiliadis E Veidal SS Barascuk N Mullick JB Clausen RE Larsen L Simonsen H Larsen DV Bay-Jensen AC Segovia-Silvestre T Leeming DJ Karsdal MA Measurement of matrix metalloproteinase 9-mediated collagen type III degradation fragment as a marker of skin fibrosis BMC Dermatol 2011 4 6 10.1186/1471-5945-11-6 21447148 PMC3072322

[B28] Gressner OA Weiskirchen R Gressner AM Biomarkers of hepatic fibrosis, fibrogenesis and genetic pre-disposition pending between fiction and reality J Cell Mol Med 2007 4 1031 1051 10.1111/j.1582-4934.2007.00092.x 17979881 PMC4401271

[B29] Hsu CC Lai SC Matrix metalloproteinase-2, -9 and -13 are involved in fibronectin degradation of rat lung granulomatous fibrosis caused by *Angiostrongylus cantonensis* Int J Exp Pathol 2007 4 437 443 10.1111/j.1365-2613.2007.00554.x 18039280 PMC2517339

[B30] Garnero P Borel O Byrjalsen I Ferreras M Drake FH McQueney MS Foged NT Delmas PD Delaissé JM The collagenolytic activity of cathepsin K is unique among mammalian proteinases J Biol Chem 1998 4 32347 32352 10.1074/jbc.273.48.32347 9822715

[B31] Gioia M Fasciglione GF Monaco S Iundusi R Sbardella D Marini S Tarantino U Coletta M pH dependence of the enzymatic processing of collagen I by MMP-1 (fibroblast collagenase), MMP-2 (gelatinase A), and MMP-14 ectodomain J Biol Inorg Chem 2010 4 1219 1232 10.1007/s00775-010-0680-8 20549272

[B32] Sukhova GK Schönbeck U Rabkin E Schoen FJ Poole AR Billinghurst RC Libby P Evidence for increased collagenolysis by interstitial collagenases-1 and -3 in vulnerable human atheromatous plaques Circulation 1999 4 2503 2509 10330380 10.1161/01.cir.99.19.2503

[B33] Charni-Ben TN Desmarais S Bay-Jensen AC Delaissé JM Percival MD Garnero P The type II collagen fragments Helix-II and CTX-II reveal different enzymatic pathways of human cartilage collagen degradation Osteoarthritis Cartilage 2008 4 1183 1191 10.1016/j.joca.2008.02.008 18403221

[B34] Murawaki Y Ikuta Y Koda M Yamada S Kawasaki H Comparison of serum 7S fragment of type IV collagen and serum central triple-helix of type IV collagen for assessment of liver fibrosis in patients with chronic viral liver disease J Hepatol 1996 4 148 154 10.1016/S0168-8278(96)80023-7 8907567

